# The chief culprit of intractable hypoxemia: a case report of rare pulmonary arteriovenous fistula complicated with giant hemangioma

**DOI:** 10.1186/s13019-024-02521-4

**Published:** 2024-02-09

**Authors:** Zhangmin Wu, Chunyu Zeng, Hongyong Wang, Weibin Shi, Xiaoli Luo

**Affiliations:** Department of Cardiology, Army Medical Center of PLA, No. 10 Yangtze River Branch Road, Yuzhong District, Chongqing, 400042 P.R. China

**Keywords:** Pulmonary arteriovenous fistula, Giant hemangioma, Hypoxemia

## Abstract

**Background:**

Pulmonary arteriovenous fistula (PAVF) is a rare disease, which can lead to the direct return of unoxidized venous blood to pulmonary veins and left heart, resulting in right-to-left shunt leading to hypoxia. Long term, the right-to-left shunt will cause severe pathophysiological changes in the patient’s body and pulmonary circulation, and the prognosis will be poor if PAVF is not treated timely.

**Case presentation:**

Here, we report the case of a 71-year-old man who presented with chest tightness and shortness of breath. After a series of examinations, PAVF and giant hemangioma were diagnosed, which are difficult to operate.Transcatheter interventional therapy was initiated. The patient recovered on the third day after operation and was discharged smoothly. During the long-term follow-up of nearly 4 years after discharge, the general condition and quality of life of the patient basically returned to normal.

**Conclusions:**

PAVF is rare but very important clinical problem. When the clinical manifestations of persistent unexplained hypoxia appear, it is necessary to fully consider the possibility of PAVF. Once the diagnosis of PAVF is clear, timely treatment is recommended to avoid deterioration of the disease and affecting the prognosis.

## Background

Pulmonary arteriovenous fistula (PAVF) is an abnormal traffic between pulmonary arteries and veins, which leads to direct return of unoxidized venous blood to pulmonary veins and left heart, resulting in a right-to-left shunt leading to hypoxia. It is relatively rare in clinics. Long term, the right-to-left shunt will cause severe pathophysiological changes in the patient’s body and pulmonary circulation, and the prognosis will be poor if it is not treated in time [1].

Herein, we reported a rare case of PAVF complicated with huge hemangioma (5 cm×2.5 cm) in an elderly man. The patient was diagnosed with"pulmonary arteriovenous fistula” more than 40 years ago, but he was not pay much attention to it. He seeks treatment just after the progressive aggravation of chest tightness, shortness of breath, hypoxia, systemic cyanosis and other symptoms. The patient was old, seriously ill, with highly difficulty and risk in treatment. Finally, he was successfully discharged after multidisciplinary escort and interventional treatment.

## Case presentation

The patient is a 71-year-old male, was admitted to the hospital on October 8, 2018, complained with chest tightness and shortness of breath after activities for more than 13 years, aggravated for 1 year. The patient’s physical strength has significantly decreased in the past 1 year. He felt short of breath while walking about 500 m, and occasionally had paroxysmal dyspnea at night. On physical examination, the temperature was 36.5 °C; respiratory rate, 20 breaths per minute; blood pressure (BP), 177/90mmHg (1 mmHg = 0.133 kPa); regular pulse rate, 104 beats/min. The patient was conscious, breathing was stable, had cyanosis of lips, face and body, no filling in the jugular vein, slightly weak breath sounds in both lungs without wet and dry rales. The heart rate was 104 beats/min, and the rhythm was regular. There were 3–4 level systolic murmurs between the 3rd and 4th ribs on the left edge of the sternum, with no pericardial rubbing sound, and no sunken edema of lower limbs.

The patient was diagnosed with arteriovenous fistula in the lower lobe of the left lung more than 40 years ago, but no attention was paid to it. In the past six years, the patient was repeatedly diagnosed with that disease and underwent surgical treatment, but he refused, and only received symptomatic treatment. Whenever the symptoms improved, he was discharged every time.

**Fig. 1:**
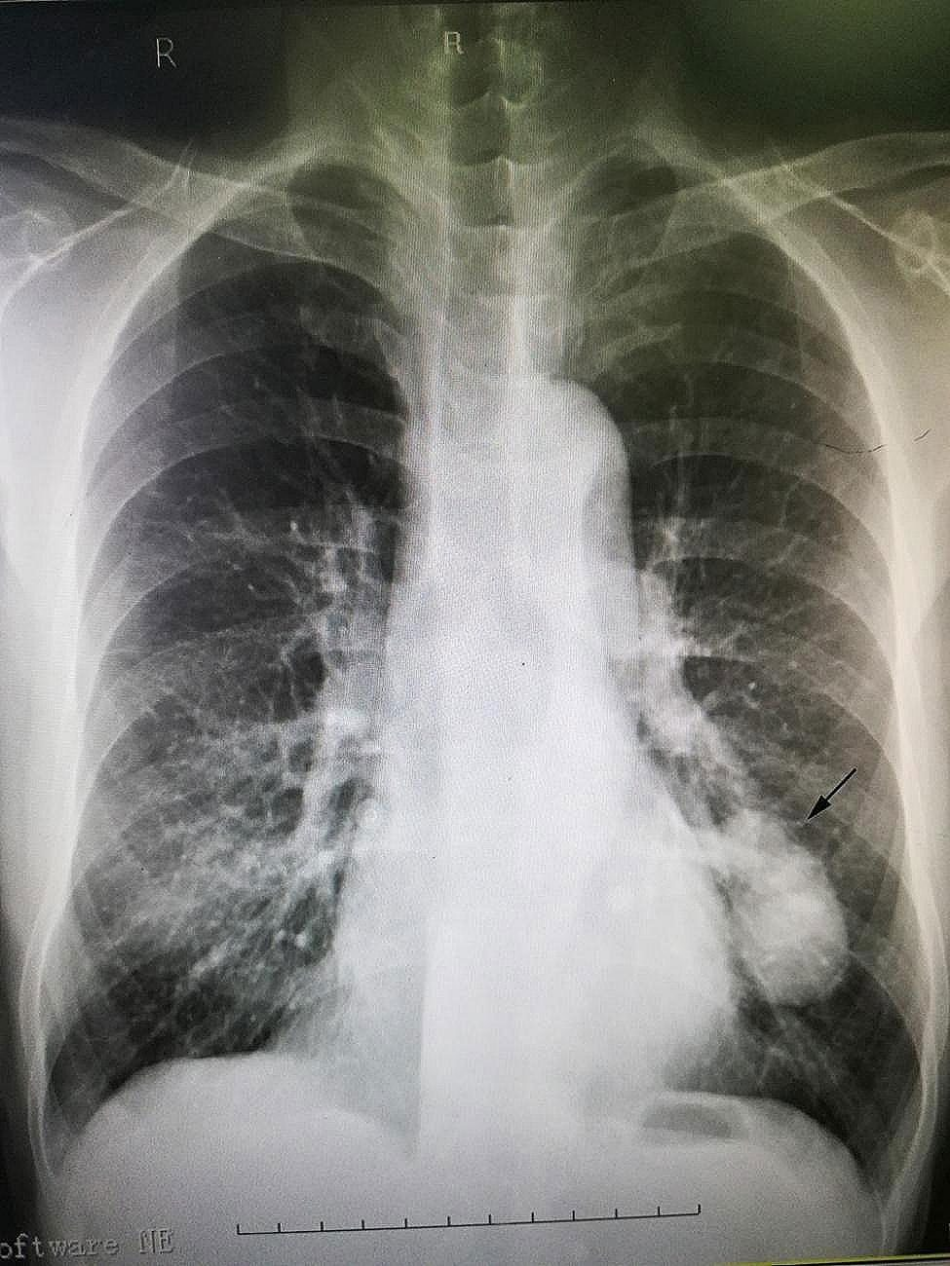
X-ray showing a mass shadow in the lower lobe of the left lung

**Fig. 2:**
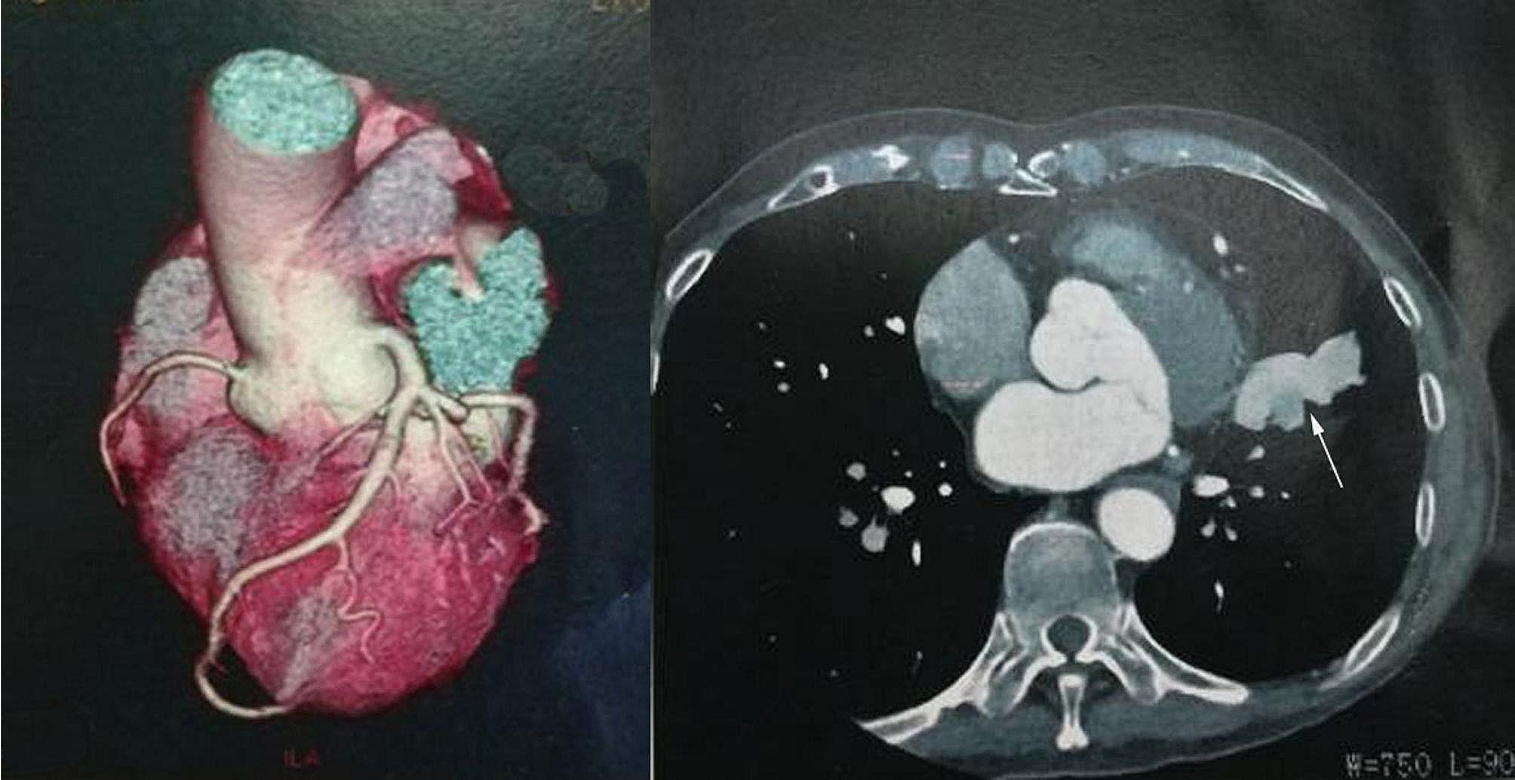
Coronary CTA showing abnormal density shadow of the left upper lobe tongue segment

**Fig. 3:**
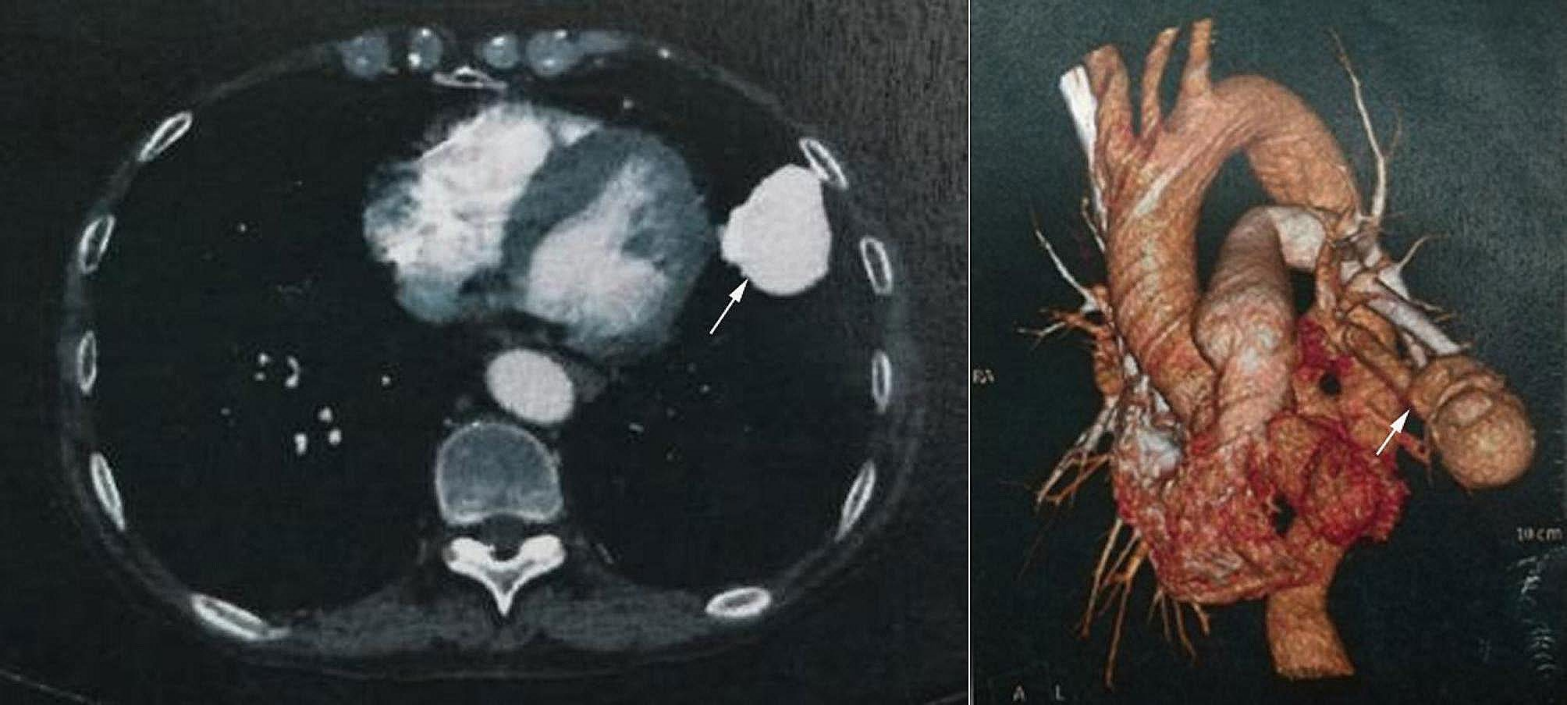
Pulmonary artery CTA showing abnormal density shadow with size about 4.7 cm×2.4 cm in the parahilar region of the left upper lobe tongue segment, considering pulmonary arteriovenous fistula

The auxiliary examination was completed after admission. Myocardial markers of injury showed creatine kinase isoenzyme (CK-MB) 1.97ug/L, myoglobin (MY0) 30.3ug/L, high-sensitivity troponin (cTNI) 0.009ug/L. B-type natriuretic peptide (BNP) 55.06 pg/ml. The fast blood gas suggested PH 7.34, PC02 35mmHg, P02 60mmHg, S02 91%, HC03- 22.7mmol/L, BE -1.3mmol/L. Blood routine examination showed white blood cell 7.15 × 10^9^/L, neutrophil ratio 76%, red blood cell 5.93 × 10^12^/L, hemoglobin 206 g/L, C-reactive protein < 0.5 mg/L. Echocardiography suggested that ventricular septal thickening (13.8 mm), left ventricular posterior wall thickening (14.1 mm), left ventricular ejection fraction was 58%. Electrocardiogram (ECG) showed sinus tachycardia, heart rate was 119 times/min, ST-T change, left atrium enlargement. Chest X-ray showed a mass shadow in the left lower lobe of the lung (Fig. 1). The coronary CTA showed calcified plaque in the proximal segment of the left anterior descending branch, with a slight stenosis of the blood vessels. And the parahilar abnormal density shadow of the left upper lobe tongue segment was attached, pulmonary arteriovenous fistula was considered (Fig. 2). Further, pulmonary artery CTA showed abnormal density shadow with a size about 4.7 cm×2.4 cm in the parahilar region of the left upper lobe tongue segment, considering pulmonary arteriovenous fistula (Fig. 3).

Preliminary diagnosis considered that left pulmonary arteriovenous fistula, coronary atherosclerotic heart disease, high blood pressure (grade 3, very high-risk group), secondary polycythemia. Provide symptomatic treatment such as anti-platelet, anti-coagulation and lowering blood pressure. The patient’s erythrocytosis was considered secondary erythrocytosis caused by long-term hypoxia of pulmonary arteriovenous fistula, and no special treatment was given temporarily.

**Fig. 4:**
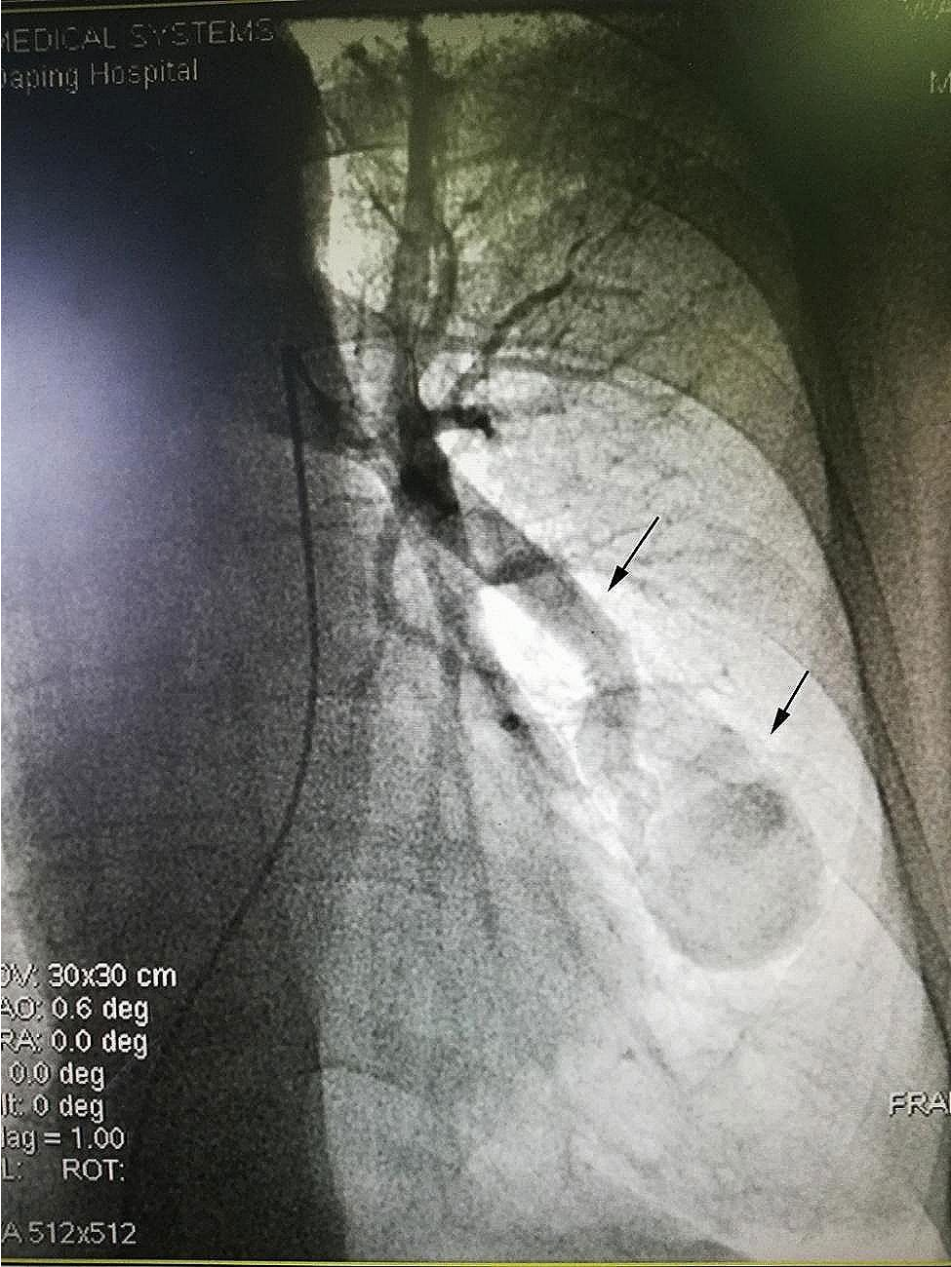
Pulmonary arteriovenous angiography showing pulmonary arteriovenous fistula with a huge hemangioma(5 cm×2.5 cm)

On the 3rd day after admission, the patient underwent the first right cardiac catheterization pulmonary arteriovenous angiography. It was found a blood flow between left pulmonary artery and pulmonary vein, with a huge hemangioma (5 cm×2.5 cm) in the middle. The narrowest part is near the hemangioma, and the left pulmonary artery is open with an inner diameter of about 1 cm. The blood flow from the distal end of pulmonary arteriovenous fistula flows back to the left pulmonary vein (Fig. 4). The patient’s left pulmonary arteriovenous fistula was large, and there was a massive hemangioma between the fistulas. It was a difficult and risky decision to block the pulmonary arteriovenous fistula at the same time, after consulting with family members, temporarily abandon the blocking the arteriovenous fistula.

On the first day after operation, that is, on the fourth day after admission, the patient developed obvious aggravation of chest tightness, shortness of breath, with sweating, cyanosis of lips and mouth. Breathing at 28 times /min, and heart rate 114 beats/min. Urgent blood gas examination showed that the pH 7.46, PC02 35 mmHg, P02 40mmHg, S02 78%%, HC03- 24.9mmol/L, BE1.4 mmol/L. The patient’s oxygen partial pressure and oxygen saturation decreased significantly, so he was given non-invasive ventilator to support ventilation, and furosemide and deacetyllanol were given at the same time to reduce the heart load. Although the oxygen flow rate was increased to 5-8 L/min and the oxygen concentration was 100%, the patient’s oxygen saturation fluctuated around 73-83%.

After organizing multi-disciplinary consultations (Cardiology, Cardiac Surgery, Thoracic Surgery, ICU, Anesthesiology), it was considered as a rare congenital disease “simple pulmonary arteriovenous fistula, type A”. The patient was old, with intractable hypoxia, poor effects of high-concentration oxygen inhalation, respiratory failure (type I), with surgical indications and no absolute contraindications. In arteriovenous fistula, the hemangioma was large, the hemangioma wall was thin and the operation risk was high. The risks of hemangioma rupture, bleeding, pneumothorax, pericardial tamponade, cardiac arrest, etc. are very likely to occur during the operation. After explaining the condition and operation to the patients and their families, they require interventional therapy. After comprehensive consideration, it is proposed that the operation should be completed in the hybrid operating room. First, medical interventional occlusion should be considered, and if necessary, surgical lobectomy should be performed.

**Fig. 5:**
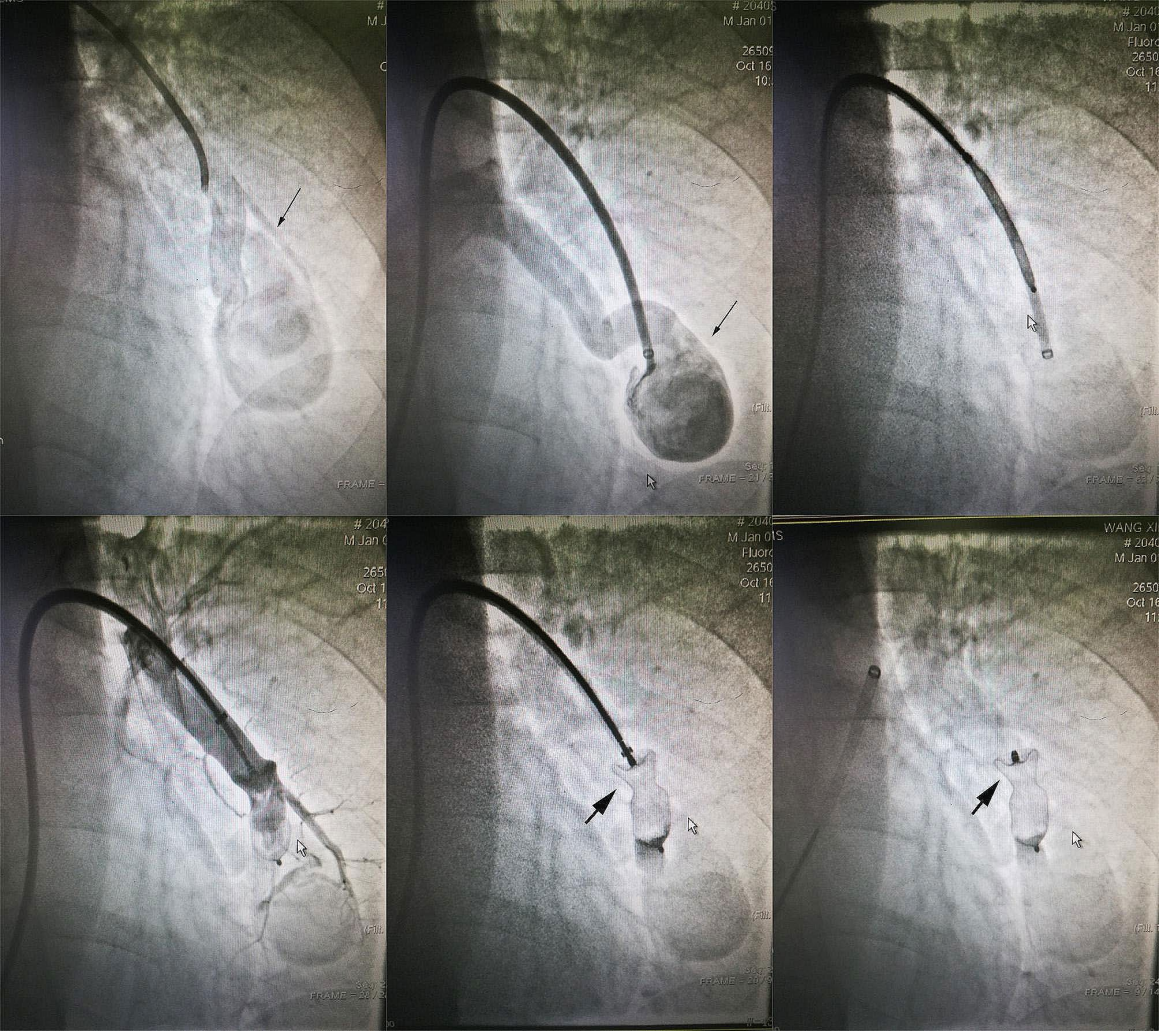
Angiography after pulmonary arteriovenous fistula occlusion showing arteriovenous shunt disappeared, and without obvious residual shunt (black solid arrow refers to pulmonary arteriovenous fistula with a huge hemangioma; black dotted arrow refers to occluder)

On the 8th day after admission, the patient underwent right cardiac catheterization and pulmonary arteriovenous angiography again. This time, the pulmonary arteriovenous fistula was successfully blocked. During the operation, it was challenging to send the occluder delivery system (9 F) along the guide wire to enter the opening of the pulmonary arteriovenous fistula through the left pulmonary artery. After that, the multifunctional angiography tube was replaced, and the hardened guidewire was replaced with a 260 mm super-slippery guidewire. With the guidance of the guidewire and support from 5MPA11 catheter, the delivery system was sent into the left pulmonary arteriovenous fistula. The delivery system was delivered to the narrowest part of the left pulmonary arteriovenous fistula by changing the 260 plus hard exchange guide wire. After exiting the guide wire and the inner core of the delivery device, the Shenzhen Xianjian patent ductus arteriosus occluder(XJFD1416)was delivered to the left pulmonary arteriovenous fistula along the delivery sheath, and then released the umbrella surface of the occluder. The delivery sheath was pulled back until the umbrella surface was close to the narrowest part at the distal end of the left pulmonary arteriovenous fistula, and then the waist of the umbrella was released further. Angiography showed that the umbrella had completely blocked the left pulmonary arteriovenous fistula, the arteriovenous shunt disappeared, and no obvious residual shunt was found (Fig. 5). In almost one minute, the patient’s blood oxygen saturation increased rapidly from about 80% under non-invasive ventilator assisted ventilation to 100% without oxygen inhalation, and the systemic cyanosis disappeared completely. After releasing the occlusion umbrella, the sheath was withdrawn and the pressure was measured along the way. The main pulmonary artery pressure, right ventricular pressure and right atrial pressure were 35/20 (26) mmHg vs. 33/18 (24) mmHg, 32/2 (21) mmHg vs. 30/2 (20) mmHg, 12/8 (10) mmHg vs. 11/8 (10) mmHg respectively before and after the operation. The operation was excellently successful. After operation, chest tightness and shortness of breath were obviously relieved, the cyanosis and heart murmur absolutely disappeared, and the peripheral blood oxygen saturation was 97 − 98% without oxygen inhalation. Rechecking blood gas showed pH 7.47, PCO_2_ 38 mmHg, PO_2_ 83mmHg, SO_2_ 97%, HCO3^−^ 27.7 mmol/L, BE^−^3.9mmol/L. In addition, the occluder was placed in the arteriovenous fistula not in heart, and the cardiac function of the patient was well before operation, complications such as heart failure did not occur after operation, so the echocardiography was not performed. After the PAVF has been blocked off, the huge hemangioma lost blood flow, additional treatment for the huge hemangioma was not needed. The patient recovered on the third day after operation and was discharged smoothly. After almost 4 years of follow-up, the patient’s general condition and quality of life essentially returned to normal.

## Discussion

Pulmonary arteriovenous fistula refers to one or more arteries in the lung that are directly connected to the vein without passing through the pulmonary circulation capillaries, and the pulmonary vessels expand and twist to form a fistula or cavernous hemangioma-like lesion, resulting in hypoxic blood in the pulmonary arteries flowing back to the left heart directly from the pulmonary veins without being oxygenated by the pulmonary capillaries, forming a right-to-left shunt [2]. Clinically, this disease is a rare disease, which is more common in teenagers and middle-aged people, and its incidence rate is low. The earlier research [3] show that it is about 2 to 3 in 100,000 people. Thanks to the improvement and popularity of examination techniques such as enhanced CT, it has been found that the incidence rate of this disease is much higher than previously estimated at 38 in 100,000 people [4]. There are two kinds of causes, congenital and acquired. Over 90% of them are caused by hereditary hemorrhagic telangiectasia (HHT) [5], which is caused by congenital vascular malformation. Some of these are caused by acquired diseases such as pulmonary inflammation, pulmonary tumor, parasites, and trauma that invade the pulmonary vessels and form fistulas between pulmonary arteries and veins. The incidence of HHT is about 0.125-0.2% [6], which is mainly characterized by multiple arteriovenous malformations, that is, the arteries and veins are directly connected due to the lack of capillaries, and the abnormally developed blood vessels expand under the action of arterial pressure, showing hemangioma-like changes. Pulmonary arteriovenous fistula mainly occurs in the lower lobe of the lung [7], and can be divided into three types of simple type, complex type and diffuse type. Among them, White et al. [8] reported that the simple type is the most common, that is, PAVF supplied by one lung segment artery and drained by a single vein, accounting for approximately 85%.

Clinical symptoms of pulmonary arteriovenous fistula lack specificity, which mainly depends on the shunt volume from right to left. If the shunt volume is low, the patient may not show obvious symptoms. When the shunt volume of unoxidized venous blood in the arterial blood exceeds 20%, symptoms related to hypoxemia may appear. Long-term “right to left shunt” may lead to organic changes of the heart and lung, hypoxia and other systemic manifestations, thus worsening the prognosis. The patient in this report has found the arteriovenous fistula of the lower left lung for over 40 years, but it was ignored because there were no obvious symptoms. Only after the symptoms appeared and worsened, which affected the patient ‘s daily activities, did he seek treatment, which not only worsened the progression of the disease, but also made the treatment more difficult, affecting the treatment effect and prognosis. Additionally, huge hemangioma (5 cm×2.5 cm) was observed in the patient, PAVF and huge hemangioma are connected continuous together, this condition is extremely rare, making it difficult and risky to block PAVF.

Imaging examination for the diagnosis of this disease includes chest X-ray, CT, CTA, DSA, MRI, etc. DSA can completely and clearly show the location, quantity, extent and morphological structure of pulmonary arteriovenous fistula, such as the diameter of fistula orifice and the amount of shunt, etc. Which is the gold standard for the diagnosis of pulmonary arteriovenous fistula [9].

Currently, the treatment methods of pulmonary arteriovenous fistula include drug-assisted therapy, surgery and interventional therapy [10]. Drug therapy is an auxiliary method of the latter two, and additional drugs include desmopressin, octreotide, estrogen, etc. Surgery is a radical measure for this disease, but it is more traumatic and not conducive to the prognosis. Percutaneous catheter interventional therapy is a new modern approach that has the benefits of small trauma and quick recovery. It can be applied to a variety of patients who can’t accept surgery. The research shows that percutaneous catheter intervention not only reduces the risks associated with traditional surgery [11, 12], but also shows that the therapeutic effect is basically similar to that of surgery [13]. At present, interventional therapy has become the first choice for the treatment of PAVF. The patient in this article finally benefited from percutaneous catheter interventional occlusion under the escort of many disciplines including the department of surgery. After the operation, he recovered quickly and was discharged safely, and showed a higher quality of life in the follow-up.

In summary, when the clinical manifestations of persistent unexplained hypoxia appear, it is necessary to fully consider whether there is the possibility of pulmonary arteriovenous fistula. Pulmonary arteriovenous fistula rarely heals on its own and tends to increase progressively. Therefore, prompt treatment is recommended to prevent deterioration of the disease and improve the prognosis once the diagnosis is clear.

## Data Availability

Data sharing is not applicable to this article as no datasets were generated or analysed during the current study.

## Abbreviations

PAVF: pulmonary arteriovenous fistula
CTA: computed tomography angiography
